# Catalytic Performance of Spherical MCM-41 Modified with Copper and Iron as Catalysts of NH_3_-SCR Process

**DOI:** 10.3390/molecules25235651

**Published:** 2020-11-30

**Authors:** Aleksandra Jankowska, Agata Chłopek, Andrzej Kowalczyk, Małgorzata Rutkowska, Marek Michalik, Shiquan Liu, Lucjan Chmielarz

**Affiliations:** 1 Faculty of Chemistry, Jagiellonian University in Kraków, Gronostajowa 2, 30-387 Kraków, Poland; a.jankowska@doctoral.uj.edu.pl (A.J.); agata.chlopek@alumni.uj.edu.pl (A.C.); kowalczy@chemia.uj.edu.pl (A.K.); rutkowsm@chemia.uj.edu.pl (M.R.); 2 Institute of Geological Sciences, Jagiellonian University in Kraków, Gronostajowa 3a, 30-387 Kraków, Poland; marek.michalik@uj.edu.pl; 3 School of Materials Science and Engineering, University of Jinan, Jinan 250022, China

**Keywords:** spherical MCM-41, NH_3_-SCR, NH_3_-SCO, copper, iron

## Abstract

Spherical MCM-41 with various copper and iron loadings was prepared by surfactant directed co-condensation method. The obtained samples were characterized with respect to their structure (X-ray diffraction, XRD), texture (N_2_ sorption), morphology (scanning electron microscopy, SEM), chemical composition (inductively coupled plasma optical emission spectrometry, ICP-OES), surface acidity (temperature programmed desorption of ammonia, NH_3_-TPD), form, and aggregation of iron and copper species (diffuse reflectance UV-Vis spectroscopy, UV-Vis DRS) as well as their reducibility (temperature programmed reduction with hydrogen, H_2_-TPR). The spherical MCM-41 samples modified with transition metals were tested as catalysts of selective catalytic reduction of NO with ammonia (NH_3_-SCR). Copper containing catalysts presented high catalytic activity at low-temperature NH_3_-SCR with a very high selectivity to nitrogen, which is desired reaction products. Similar results were obtained for iron containing catalysts, however in this case the loadings and forms of iron incorporated into silica samples very strongly influenced catalytic performance of the studied samples. The efficiency of the NH_3_-SCR process at higher temperatures was significantly limited by the side reaction of direct ammonia oxidation. The reactivity of ammonia molecules chemisorbed on the catalysts surface in NO reduction (NH_3_-SCR) and their selective oxidation (NH_3_-SCO) was verified by temperature-programmed surface reactions.

## 1. Introduction

Development of mesoporous silica materials in 1990s [1,2] was undoubtedly one of the most specular challenges in materials sciences. Due to their huge surface area, pore volume, and uniform porosity in the mesopores range there was especially great hope for the application of these materials in catalysis and adsorption. It was believed that mesoporous silica materials would play a similar role in catalysis as zeolites. Unfortunately, mesoporous silica materials, in contrast to zeolites, are composed of amorphous silica what limit their thermal and mechanical stability [3]. Introduction of guest elements (e.g., Al^3+^) into mesoporous silica materials may results in the formation of surface acid sites and ion-exchange properties. However, in contrast to zeolites, there is a high heterogeneity in the strength of such acid sites and ion-exchange potential is significantly limited [4]. Various methods of catalytically-active components’ deposition into mesoporous silica, including grafting of metalorganic compounds [5], generation of ion-exchange properties by anchoring of organic functional groups [6], template ion-exchange method [7], or impregnation [8], have been applied. Deposition of catalytically-active metals by grafting methods, e.g., molecular designed dispersion (MDD), results in highly-dispersed metal species; however, this method is rather expensive and only limited amounts of metal species can be deposited into silica support [8]. Similarly, relatively high cost and limited metal loadings are the main drawbacks of the method based on anchoring of functional groups with ion-exchange properties on the silica surface followed by metal deposition by ion-exchange method [9]. Deposition of catalytically-active metals by template ion-exchange (TIE) method is based on exchange of cationic surfactants in freshly prepared mesoporous silica materials for metal cations [10]. The TIE method is very promising because the catalysts with relatively large metal loading can be obtained by relatively simple and cheap procedure. However, this method needs individual optimization for each deposited metal to control its form and aggregation [11]. Impregnations are the simplest methods but in these cases the metal deposition in the form of uniform species is out of control [12]. An interesting option is the introduction of catalytically-active metal species by co-condensation method during mesoporous silica formation [13]. This relatively simple and cheap method, depending on the synthesis conditions, may result in quite uniform distribution of metal species in silica matrix. Moreover, metal species incorporated into silica walls are strongly stabilized against sintering under catalytic reaction conditions.

Mesoporous silica MCM-41 in the spherical form seems to be very promising catalytic support [14]. In this case mesoporous silica is in the form of small spheres with typical diameter below one micrometer and therefore the average distance from the outer surface of the silica spheres to active sites located inside pores is shorter comparing to classical MCM-41. Thus, the possible limitation of the overall reaction rate by internal diffusion of reactants is less likely in the case of spherical MCM-41 than for classical MCM-41 support. 

The presented studies include the synthesis of spherical MCM-41 with copper and iron species incorporated into amorphous silica matrix and verification their catalytic performance in the selective catalytic reduction of NO with ammonia (NH_3_-SCR). The efficiency of this process at higher temperatures is limited by the side reaction of direct ammonia oxidation by oxygen present in flue gases. Therefore, also the activity of the catalysts in the reaction of selective ammonia oxidation (NH_3_-SCO) was studied and compared with their catalytic activity in the process of NO reduction with ammonia.

## 2. Results and Discussion

The spherical MCM-41 samples containing various content of copper or iron incorporated into the silica walls were obtained by surfactant directed co-condensation method. The following sample codes, *X*Cu-MCM-41 and *X*Fe-MCM-41, are used, where *X* indicates the intended molar Si/Cu or Si/Fe ratios in the studied samples.

### 2.1. Characterization of Catalysts 

**Figure 1:**
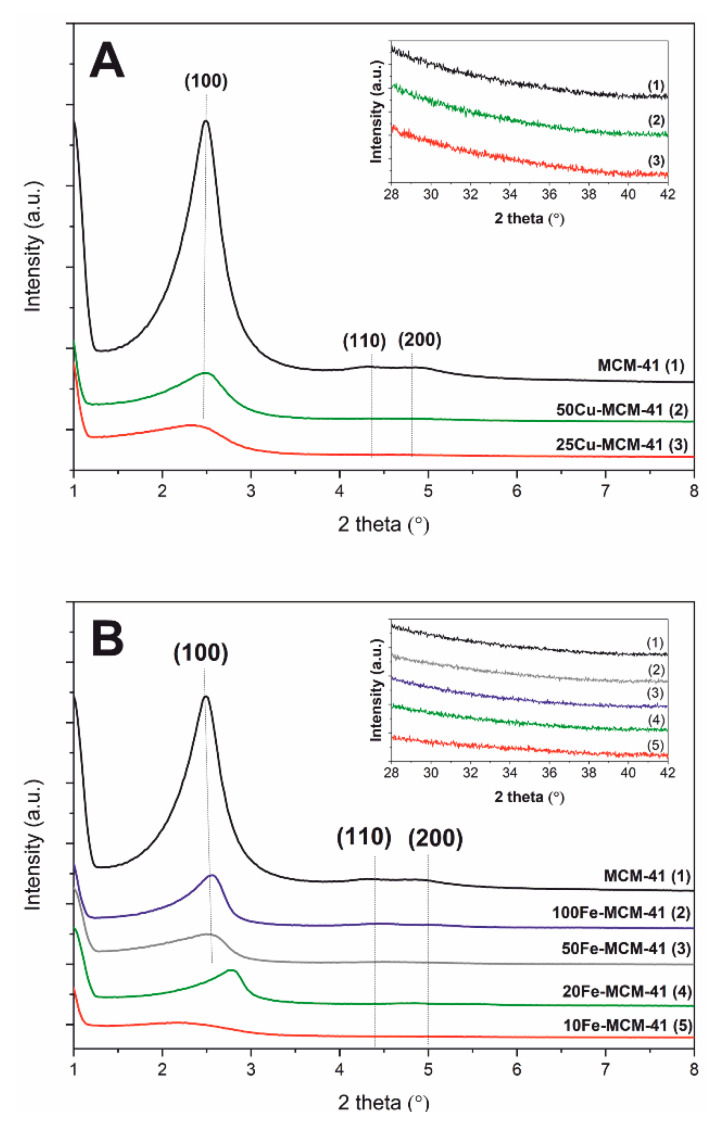
XRD patterns of the copper (**A**) and iron (**B**) containing spherical MCM-41 samples.

X-ray diffraction patterns of spherical MCM-41 and its modifications with copper and iron are presented in Figure 1. Three reflections—(100), (110), and (200)—typical of the MCM-41 hexagonal porous structure [15], present in diffractogram of spherical MCM-41 (Figure 1A), indicate successful synthesis of this sample. Incorporation of copper into spherical silica walls resulted in a decrease of these reflections intensity (Figure 1A), which is indicative of the less ordered porous structure of these samples. Similar effect was observed for spherical MCM-41 containing iron incorporated into silica walls (Figure 1B). Moreover, the shift of the (100) reflection into higher 2 theta values for the sample modified with iron indicates decrease in the average pore diameter. It should be also noted that any reflection characteristic of copper and iron oxides was not found in diffractograms of the studied silica samples modified with transition metals (inserts in Figure 1A,B). Thus, the size of crystallites of these metal oxides, if present in the samples, is below the detection level of XRD method.

**Figure 2:**
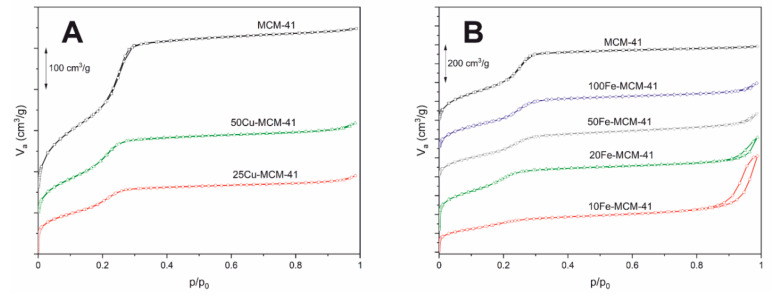
N_2_ adsorption-desorption isotherms of the spherical MCM-41 samples containing Cu (**A**) and Fe (**B**).

**Figure 3:**
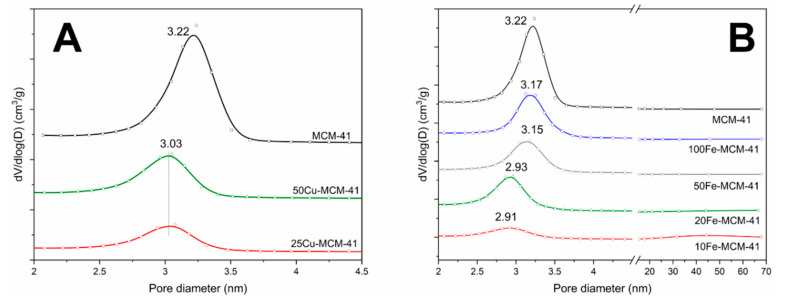
Pore size distributions of the of the spherical MCM-41 samples containing Cu (**A**) and Fe (**B**).

N_2_-sorption isotherms and profiles of pore size distributions (PSD) of the samples are presented in Figure 2 and Figure 3, respectively. Moreover, textural parameters of the studies samples are shown in Table 1. The isotherm recorded for the spherical MCM-41 sample is classified as type IV according to the IUPAC classification. This type of isotherm is characteristic of mesoporous materials, such as MCM-41 [16]. The characteristic steep of nitrogen uptake at relative pressure of 0.20–0.40 indicates capillary condensation of N_2_ molecules in the mesopores of spherical MCM-41 (Figure 2A). Incorporation of copper into MCM-41 silica walls resulted in a decrease of nitrogen uptake step indicating limitation of mesopore volume in these samples. Similar effect is observed for the samples modified with iron (Figure 2A). In this case the correlation between iron content (Table 1) and intensity of nitrogen uptake step was found, indicating that incorporation of iron into the silica walls decreases mesopore volume. In isotherms of the transition metal containing samples an increase in nitrogen adsorption volume was observed at p/p_0_ relative pressure above 0.85. This effect, more distinct for the samples with the highest iron loadings, 10Fe-MCM-41 and 20Fe-MCM-41, is possibly attributed to the pore structure changes. Profile of pore size distribution (PSD) of spherical MCM-41, presented in Figure 3A, consists of symmetric maximum centered at about 3.22 nm. Incorporation of copper into silica walls decreased intensity of this maximum but also resulted in its shift to about 3.03 nm, indicating a significant decrease in the size of mesopores in these samples. Similar effect was observed for iron containing spherical MCM-41 (Figure 3B). In this case correlation between iron loading and decrease of the mesopore diameter was found. Textural parameters of the studied samples, presented in Table 1, show a gradual decrease in their BET (Brunauer–Emmett–Teller) specific surface areas and pore volumes with the increasing copper or iron loadings. Incorporation of copper into the silica walls more significantly decreased textural parameters comparing to introduction of iron. The mechanical stability of the selected samples, 50Cu-MCM-41 and 20Fe-MCM-41, was analyzed by their squeezing in a hydraulic press with the force of 3 tons. The specific surface area (S_BET_) and pore volume (PV) determined for the samples treated in this way are shown in Table 1 (values in brackets). It can be seen that in the case of iron containing sample small increase in textural parameters is observe. The S_BET_ values increased by about 5%, while PV by about 2%. Taking into account that the accuracy of this method is around 10% it could be concluded that there in not significant changes in textural parameters of this sample, which is stable under pressing conditions. The S_BET_ and PV values determined for the copper containing sample decreased after pressing by about 22% and 27%, respectively. Thus, it seems that incorporation of copper into the spherical MCM-41 walls results in decreasing of its mechanical stability.

**Figure 4:**
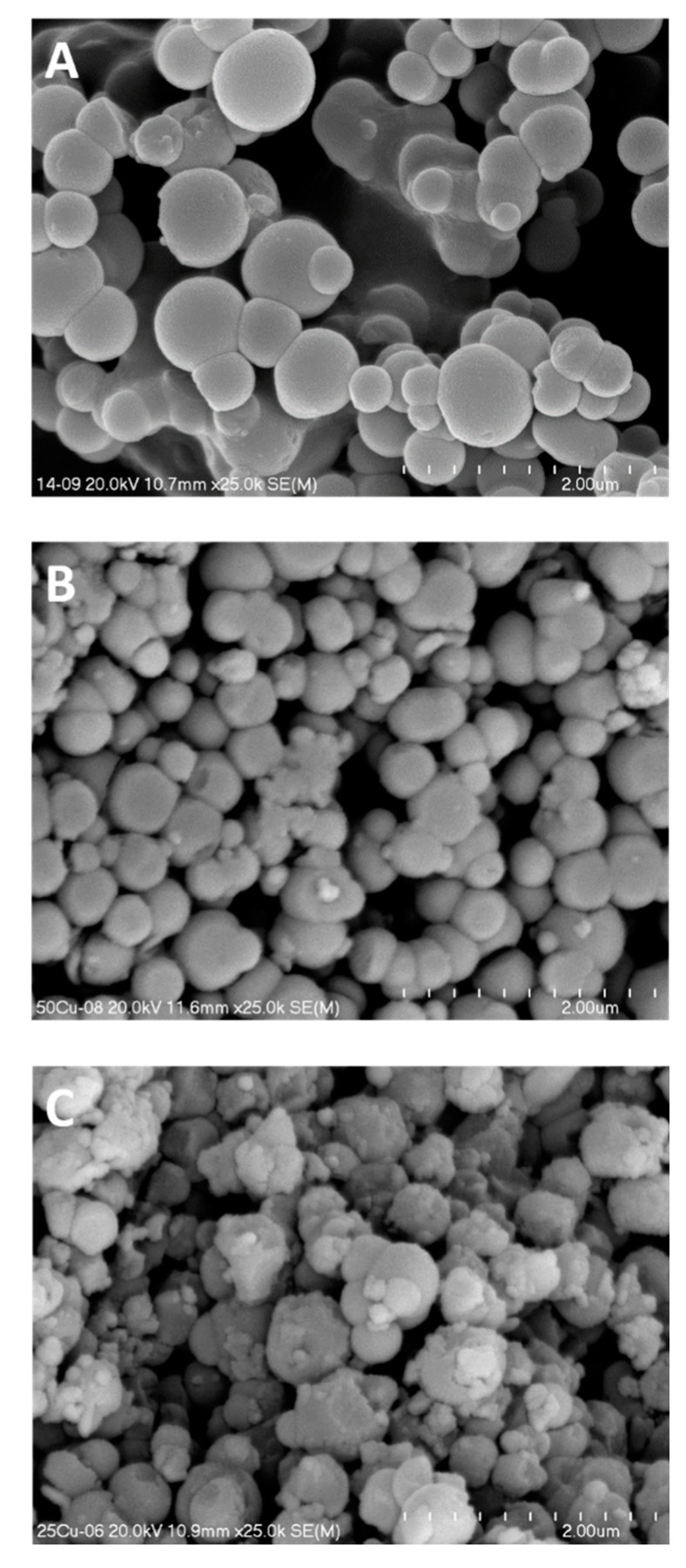
SEM micrographs of spherical Si-MCM-41 (**A**) and its modifications with copper—50Cu-MCM-41 (**B**) and 25Cu-MCM-41 (**C**).

**Figure 5:**
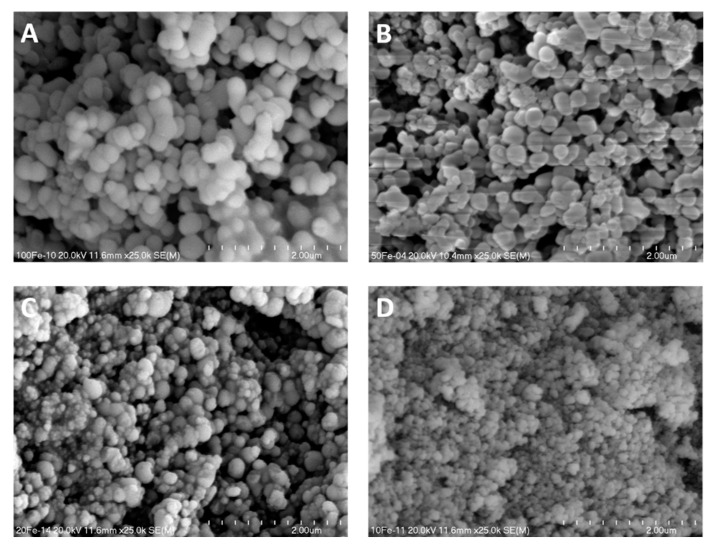
SEM images of spherical MCM-41 modified with iron: 100Fe-MCM-41 (**A**), 50Fe-MCM-41 (**B**), 20Fe-CM-41 (**C**), and 10Fe-MCM-41 (**D**).

The SEM micrographs of pure silica spherical MCM-41 and its modifications with copper are presented in Figure 4. The silica spheres in pure silica materials are in the range from about 300 to 600 nm (Figure 4A). Incorporation of copper into spherical MCM-41 resulted in decrease of silica spheres to about 150–500 nm in the case of 50Cu-MCM-41 (Figure 4B), while for the 25Cu-MCM-41 more significant contribution of smaller spheres and irregular agates of amorphous silica sticked to larger spheres can be observed (Figure 4C). Similar effect of silica spheres size decrease and spherical shape disordering was observed for the samples with the increasing iron loading (Figure 5). The spheres of the samples, especially those with higher iron content, are sticked together forming large inter-spheres pores. The results of SEM analysis are in line with the analysis of textural properties, which showed nitrogen adsorption step at p/p_0_ above 0.85 for the samples with the highest iron-loadings, 10Fe-MCM-41 and 20Fe-MCM-41, possibly related to the presence of inter-spherical pores (Figure 2). Also pore volume for these samples is significantly larger than that measured for other materials of this series (Table 1).

The copper and iron content as well as the molar Si/Cu and Si/Fe ratios in the samples are presented in Table 1. As it can be seen the real Si/Cu and Si/Fe ratios are slightly lower than intended values, indicating that transition metals are preferentially incorporated into the silica walls. The only exception is the 20Fe-MCM-41 sample, in which the real Si/Fe molar ratio is slightly higher (21.4) comparing to the intended value of 20.

**Figure 6:**
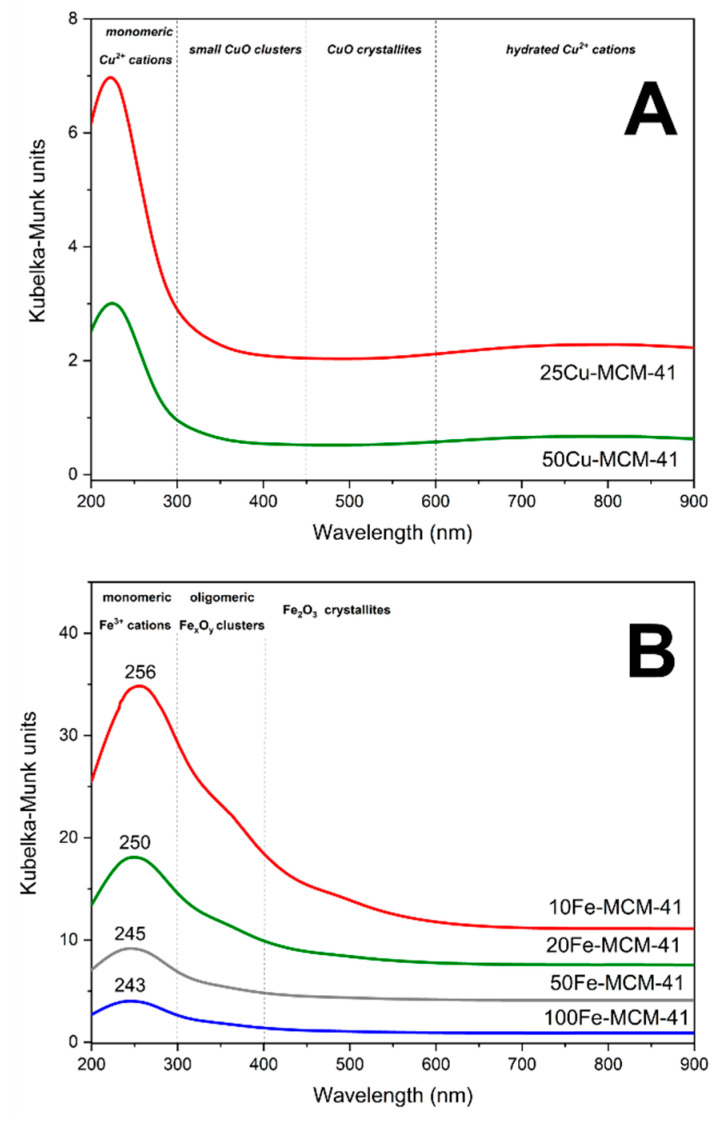
UV-vis-DR spectra of the spherical MCM-41 based samples modified with copper (**A**) and iron (**B**).

The coordination and aggregation of copper and iron species present in spherical MCM-41 was analyzed by UV-vis-DR spectroscopy. In the spectra recorded for copper modified silicas the intensive bands centered at 225 nm (Figure 6A), indicating the presence monomeric copper cations interacting with oxygen of silica matrix (O^2−^→Cu^2+^) [7,17]. The presence of such monomeric copper cations as dominating metal species in the 50Cu-MCM-41 and 25Cu-MCM-41 samples is proved by the broad bands at about 760–780 nm attributed to d-d transition in the monomeric Cu^2+^ ions in pseudo-octahedral coordination, e.g., Cu(H_2_O)_6_^2+^ [7,17]. Small shoulders at above 300 nm indicate the presence of oligomeric copper oxide species [7,17]. Thus, monomeric copper cations dominate in the 50Cu-MCM-41 and 25Cu-MCM-41 samples. Moreover, small contribution of oligomeric copper oxide species in these samples was identified.

The UV-vis-DR spectra of the iron containing samples, presented in Figure 6B, consist of intensive maximum centered at about 243–256 nm and shoulder above 300 nm, indicating the presence of monomeric Fe^3+^ cations and small oligomeric FeO_x_ clusters, respectively [8,9,18]. Moreover, the shoulders at above 400 nm, observed in spectra of the samples with the highest iron loadings, 10Fe-MCM-41 and 20-MCM-41, indicate the presence of crystalline Fe_2_O_3_ particles [8,9,18]. Thus, an increase in iron loading resulted in deposition, apart from monomeric Fe^3+^ cations, also more aggregated iron oxide species. It should be also noted that for the samples with increasing iron content the maximum related to the presence of monomeric iron cations was shifted to higher values of wavelength, from 243 nm for 100Fe-MCM41 to 256 nm for 10Fe-MCM-41 (Figure 6B). It has been reported that the maximum below 250 nm indicates the presence of Fe^3+^ cations in tetrahedral coordination, while maximum above 250 nm is assigned to Fe^3+^ cations in octahedral coordination [19]. It seems that in the case of the studied samples, there is a superposition of both these maxima, however in the samples with the lower iron loading the contribution of monomeric iron cations in tetrahedral coordination is more significant than for the samples with higher iron content.

**Figure 7:**
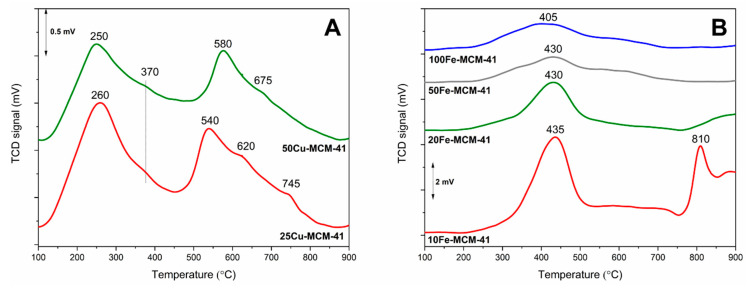
Results of H_2_-TPR studies of the spherical MCM-41 based samples doped with Cu (**A**) and Fe (**B**).

Reducibility of the transition metals species present in catalysts is a very important feature, which often influences their activity in various processes, including NH_3_-SCR and NH_3_-SCO [20,21]. Temperature-programed reduction with hydrogen as reducing agent (H_2_-TPR) was used for the reducibility analysis of the copper and iron species present in the spherical MCM-41 samples (Figure 7). The reduction profiles of the copper doped samples, 50Cu-MCM-41 and 25Cu-MCM-41, consist of the intensive maxima at lower temperature, 250–260 °C, and at higher temperature, 540–580 °C (Figure 7A). For both peaks additional shoulders from the side of higher temperatures are present. In general, the low-temperature maximum is assigned to the reduction of Cu^2+^→Cu^0^ in aggregated copper oxide species and Cu^2+^→Cu^+^ in monomeric copper cations, while the high-temperature peak to the second step of monomeric cations reduction of Cu^+^→Cu^0^ [7]. Probably the main peaks are related to the reduction of easy accessible surface copper species, while the shoulders are assigned to the reduction of copper species occluded in the silica walls of MCM-41. Comparison of the surface areas integrated under low- and high-temperature maxima in the reduction profiles of the copper containing samples leads to the conclusion that in the case of 50Cu-MCM-41 about 76% of copper was deposited in the form of monomeric Cu^2+^ cations, while 24% in the form of aggregated copper oxide species. Similar results were obtained for 25Cu-MCM-41, which according to the analysis contains 78.5% of copper in the form of monomeric cations and 21.5% in the form of aggregated copper oxide species. It must be underlined that the analysis of copper species distribution was done assuming that copper in all species present in the samples exists as Cu^2+^ prior to the H_2_-TPR runs. Thus, the presented contributions of monomeric and aggregated copper species are not precise values but rather rough estimation. However, such estimation clearly shows that copper is present in the samples mainly in the form of monomeric copper cations, irrespective of metal loading. The results of H_2_-TPR studies are in full agreement with the UV-Vis DRS analysis (Figure 6A), which proved a dominant contribution of monomeric copper cations in the 50Cu-MCM-41 and 25Cu-MCM-41 samples.

Reduction profiles of the iron containing samples, presented in Figure 7B, consist of broad maxima located at about 405–435 °C with tails from the side of higher temperatures and, in the case of the samples with the highest iron loadings, maxima above 750 °C. The detailed analysis and discussion of iron species reduction in MCM-41 was done by Mokhonoana and Coville [15] as well as Kiatphuengporn et al. [22]. The maxima at about 405–435 °C are related to the reduction of Fe_2_O_3_→Fe_3_O_4_, the shoulder from the side of higher temperatures to the reduction of Fe_2_O_3_→FeO + Fe. The hydrogen consumption observed in the reduction profiles of 10Fe-MCM-41 and 20Fe-MCM-41 above 750 °C is assigned to the FeO→Fe^0^ reduction [15,22]. It should be noted that the main reduction maximum is slightly shifted to higher temperatures for the samples with higher iron loadings. Similar correlation is observed for the temperatures of the reduction process beginning. As it was shown by UV-Vis DRS analyses, in the samples with higher iron loading there is more significant contribution of the aggregated iron oxide species (Figure 6B). Thus, it could be postulated that more aggregated iron oxide species are more resistant for reduction (Fe_2_O_3_→Fe_3_O_4_). The shoulder in the temperature range of 550–750°C is possibly related to the Fe_2_O_3_→FeO + Fe reduction [15,22]. The last stage of iron species reduction (FeO→Fe^0^) is observed above 750 °C only for the samples with the highest iron loading and with the significant contribution of aggregated iron oxide species. The absence of such high temperature peaks in the reduction profiles of the samples with the lower iron loadings indicates that highly dispersed FeO species are more effectively stabilized by silica matrix against reduction to metallic iron than more aggregated iron oxide species.

**Figure 8:**
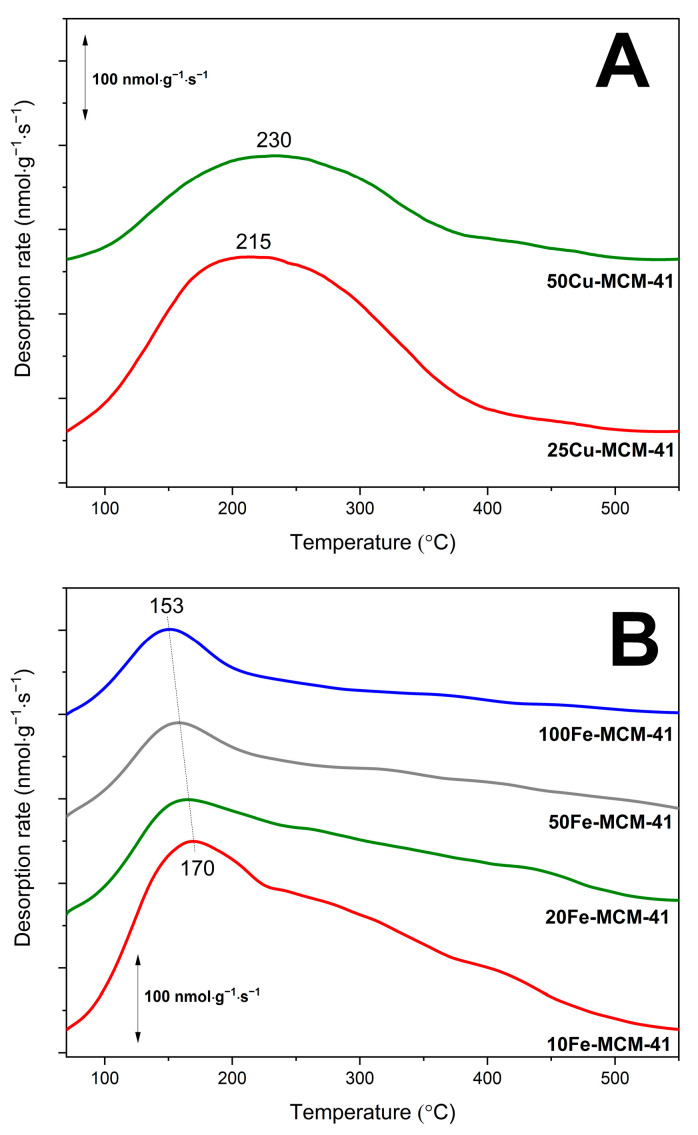
NH_3_-TPD profiles of spherical MCM-41 modified with copper (**A**) and iron (**B**).

Surface acidity of the mesoporous silica samples modified with transition metals was studied by temperature-programmed desorption of ammonia (NH_3_-TPD). Pure silica spherical MCM-41 does not exhibit the presence of acid sites. Thus, ammonia adsorption on the samples is related only to deposited transition metals species by possible accommodation of free electron pair of ammonia molecule by unoccupied *d* orbital of transition metal (NH_3_→Cu^2+^ or NH_3_→Fe^3+^). Therefore, the NH_3_-TPD measurements are very useful for determination of the surface accessible transition metal cations available for catalysis. On the other side, ammonia is not only probe molecule in NH_3_-TPD but also reactant in NH_3_-SCR and NH_3_-SCO reactions. Taking into account that the majority of the reported mechanisms of NH_3_-SCR and NH_3_-SCO reactions includes ammonia chemisorption and activation on the catalyst surface [21,23] the results of NH_3_-TPD studies are important for understanding the role of deposited transition metals in ammonia conversion. The ammonia desorption profiles of the copper containing samples, presented in Figure 8A, consist of broad maxima centered at 215–230 °C with tails from the side of higher temperatures. Such shapes of desorption profiles indicate a significant heterogeneity of ammonia bounding to copper centers. As it was proved by UV-vis DRS (Figure 6A) and H_2_-TPR (Figure 7A), copper in the 50Cu-MCM-41 and 25Cu-MCM-41 samples was deposited mainly in the form of monomeric cations and therefore such broad ammonia desorption peaks may indicate that not only form of deposited copper species but also their location and surrounding in silica matrix possibly modify their interactions with ammonia.

Ammonia desorption profiles of the iron containing samples, presented in Figure 8B, are spread from 80 to 550°C indicating very high heterogeneity in the strength of ammonia bounding by iron species. The main ammonia desorption peak is centered in the range of 153–170 °C depending on the iron content in spherical MCM-41. For the samples with the lower iron content this maximum is located at slightly lower temperatures comparing to silicas with higher iron loadings. As it was shown by UV-vis DRS studies, for the samples with increasing iron loading the contribution of aggregated iron oxide species increased (Figure 6B). Thus, it could be postulated that chemisorbed ammonia molecules are slightly more effectively stabilized on aggregated iron oxide species comparing to monomeric Fe^3+^ cations or less aggregated iron species. Of course, this is only hypothesis that should be verified in the future.

The surface concentrations (C_a_) and surface densities (D_a_) of acid sites in the samples, determined from the NH_3_-TPD measurements, are presented in Table 1. It was assumed that one ammonia molecule is chemisorbed on one acid site and therefore the concertation of chemisorbed ammonia is equal to the number of surface acid sites. Moreover, the molar ratio of ammonia chemisorbed quantity on the samples surface to metal loadings (NH_3_/Cu-Fe) are shown in this table. Assuming that one ammonia molecule is bounded to one metal cation and that ammonia can be bounded only to the metal cations exposed on the sample surface, the ratio of such surface metal cations to total metal loading in the samples can be determined. These values indicate the contribution of surface accessible metal cations that act as catalytically-active sites in the studied reactions. As it can be seen from Table 1, the NH_3_/Cu and NH_3_/Fe molar ratios decrease with an increase in transition metal loadings.

### 2.2. Catalytic Studies of NH_3_-SCR and NH_3_-SCO

**Figure 9:**
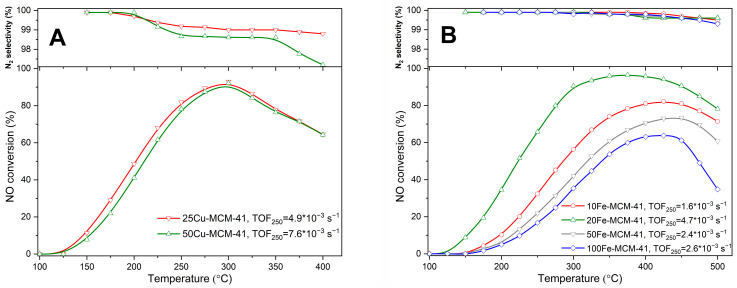
Temperature dependence of NO conversion and N_2_ selectivity in SCR of NO with NH_3_ for spherical MCM-41 modified with copper (**A**) and iron (**B**).

Spherical MCM-41 modified with copper and iron was studied as catalysts of the selective catalytic reduction of NO with ammonia (NH_3_-SCR) and selective catalytic oxidation of ammonia (NH_3_-SCO). In the presence of copper containing catalysts, 50Cu-MCM-41 and 25Cu-MCM-41, the NO reduction with ammonia (NH_3_-SCR) started at relatively low temperature of 125 °C and increased to the level of about 90% at 300 °C (Figure 9A). At higher temperatures efficiency of the NO conversion decreased due to the side process of direct ammonia oxidation by oxygen present in the reaction mixture. The activity of catalyst with the larger copper loading, 25Cu-MCM-41, is only slightly higher comparing to 50Cu-MCM-41. Such small difference in catalytic activity of these catalysts is surprising taking into account that copper content in 25Cu-MCM-41 is more than twice higher comparing to 50Cu-MCM-41. Thus, possibly copper species in these catalysts present different activity in the NO reduction with ammonia. To more precisely evaluate activity of the copper species in these catalysts turn-over-frequency (TOF) for the reaction at temperature 250 °C was determined. It was assumed that the role of catalytically-active sites play surface accessible copper cations, determined by NH_3_-TPD measurements (Table 1). The TOF value for 50Cu-MCM-41 is significantly higher than for 25Cu-MCM-41 (Figure 9A), indicate ng that average activity of separate active site is higher for the sample with the lower copper content. This result is surprising taking into account very similar types of deposited copper species in both these samples determined by UV-vis DRS (Figure 6A) and H_2_-TPR (Figure 7A). May be slightly better reducibility of copper species in the low-temperature range observed for 50Cu-MCM-41 (Figure 8A) is one of reasons explaining the higher activity of copper active sites in this sample. It should be noted that the copper containing catalysts presented very high selectivity to nitrogen, which is a desired reaction product (Figure 9A).

The results of the catalytic tests in the NH_3_-SCR process for iron containing catalysts, presented in Figure 9B, show a very strong dependence of the catalytic activity and iron content in the samples. The catalytic activity increases with the increasing iron loading in the following order: 100Fe-MCM-41 < 50Fe-MCM-41 < 20Fe-MCM-41. The catalyst with the highest iron loading, 10Fe-MCM-41, presented the lowest catalytic activity in this series of the samples. The results of UV-vis DRS studies (Figure 6B) and nitrogen sorption (Table 1) show that an increase in iron loading resulted in increasing contribution of aggregated iron oxide species and decrease in BET surface area and therefore limited accessibility of iron cations for catalytic reaction. In order to determine average catalytic activity of surface accessible iron cations the TOF values were determined for the reaction at 250 °C. It was assumed that each ammonia chemisorbed molecule determined by NH_3_-TPD measurements indicates one surface accessible iron cation, which plays a role of catalytically-active site. The TOF values, presented in Figure 9B, show that the most active sites are pressed in 20Fe-MCM-41, which is the most effective catalyst of this series. Lower activity presented iron sites in the samples with the lower iron loadings, 50Fe-MCM-41 and 100Fe-MCM-41. The active sites of lower average activity were present in the catalyst with the highest iron loading, 10Fe-MCM-41. Thus, it could be postulated that the aggregation of iron species is very important for their catalytic activity in the NH_3_-SCR process. It seems that highly dispersed iron species, mainly monomeric cations are less active than small aggregated iron species, possibly metal oxide oligomers. On the other side an increase in aggregation of such species results in less catalytically-active sites. Also the optimal ratio between highly dispersed and more aggregated species as factor determining activity of the catalysts cannot be excluded. It has to be stress that there are only hypotheses that should be verified in the future. In the case of the most active catalyst of this series, 20Fe-MCM-41, the NO conversion started at about 125 °C and the conversion above 90% was obtained in the range of 300–450 °C (Figure 9B). The commercial catalysts for the NH_3_-SCR process, based on V_2_O_5_-TiO_2_ metal oxide system, operate in the range of 300–400 °C [23], thus the studied catalyst extended temperature window of the effective catalytic operation from the side of higher temperatures by about 50 °C. The efficiency of the NO conversion in the high temperature range is limited by the side process of direct ammonia oxidation. The selectivity to nitrogen for 20Fe-MCM-41 and also other catalysts of this series is very high in the studied temperature range, what is very promising.

**Figure 10:**
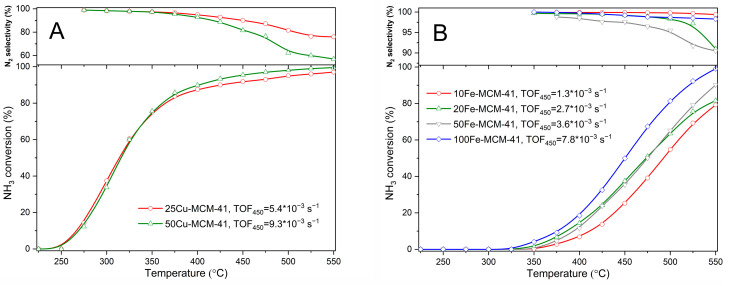
Temperature dependence of NH_3_ conversion and N_2_ selectivity in SCO of NH_3_ for spherical MCM-41 modified with copper (**A**) and iron (**B**).

The high temperature reduction of NO with ammonia is limited by direct ammonia oxidation by oxygen present in the reaction mixture. Therefore, the activity of the studied catalysts in the process of selective ammonia oxidation (NH_3_-SCO) was verified (Figure 10). Ammonia oxidation started at about 250 °C and sharply increased to about 400 °C reaching the ammonia conversion on the level 85–87% (Figure 10A). An increase in the ammonia conversion was significantly less intensive at higher temperatures. For the catalysts, 50Cu-MCM-41 and 25Cu-MCM-41, the ammonia conversion profiles are very similar despite the copper content is significantly different. Such differences can be explained by different catalytic activity of copper species present in the samples. The TOF values determined for the copper containing samples in the NH_3_-SCO reaction conducted at 450 °C are presented in Figure 10A. Nitrogen was the main product of ammonia oxidation in the whole studied temperature range—below 350 °C nitrogen was the only reaction product but at higher temperatures the selectivity to N_2_ gradually decreased reaching at 550 °C the level of 57 and 76% for 50Cu-MCM-41 and 25Cu-MCM-41, respectively. Comparison of the NH_3_-SCR (Figure 9A) and NH_3_-SCO (Figure 10A) results clearly shows that the NO reduction with ammonia dominates below 300 °C, however at higher temperatures significant amounts of ammonia are oxidized and therefore, due to its shortage, the efficiency of the NO reduction in the NH_3_-SCR process decreased.

Ammonia oxidation started at about 325–350 °C in the presence of Fe-modified MCM-41 (Figure 10B), so at temperatures significantly higher than in the case of copper containing catalysts (Figure 10A). In this series of the catalysts the compete ammonia oxidation was obtained in the studied temperature range only in the presence of 100Fe-MCM-41, so the catalyst with the lowest iron content. It is surprising, however it should be taken into account that not metal loading but their surface accessibility and reactivity determines the activity of the catalysts. The TON values for the catalytic reaction conducted at 450 °C (Figure 10B), indicating the average catalytic activity of individual surface accessible iron cation, show that their catalytic activity increases with decreasing iron content in the samples. Thus, less aggregated iron species, possibly monomeric Fe^3+^ cations, are significantly more active in ammonia oxidation comparing to aggregated iron oxide species. It should be noted that nitrogen is the main product of ammonia oxidation and N_2_ selectivity for the all catalysts of this series was above 90% in the studied temperature range (Figure 10B). Comparison of the results of NH_3_-SCR (Figure 9B) and NH_3_-SCO (Figure 10B) tests shows that the NO reduction with ammonia is not affected by direct ammonia oxidation to about 350 °C. The role of ammonia oxidation reaction gradually increased above this temperatures resulting in decreased efficiency of the NH_3_-SCR process.

### 2.3. Temperature-Programmed Surface Reactions

**Figure 11:**
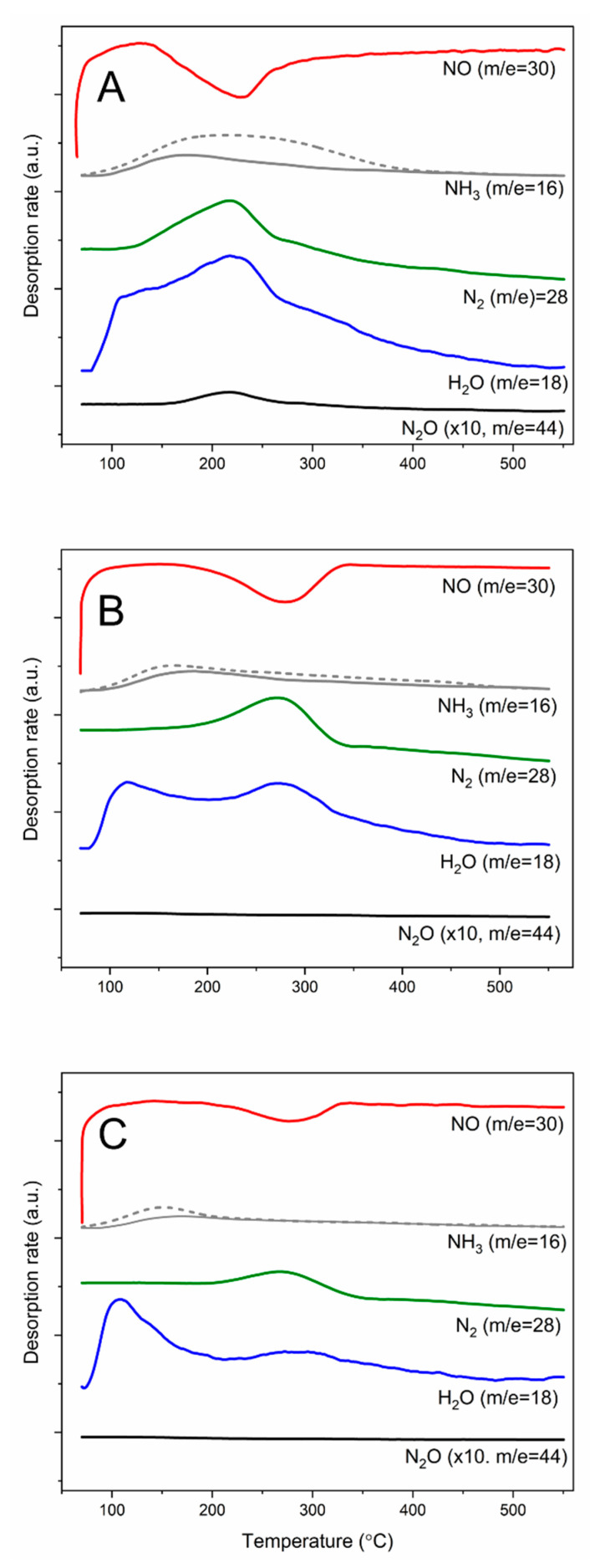
Results of NH_3_-SCR temperature-programmed surface reaction for 25Cu-MCM-41 (**A**), 20Fe-MCM-41 (**B**), and 100Fe-MCM-41 (**C**).

Because majority of NH_3_-SCR [23] and NH_3_-SCO [21] mechanisms postulates ammonia chemisorption and activation on the catalyst surface the studies of ammonia molecules reactivity adsorbed on the selected catalysts in both reactions were done. Figure 11 presents the results of such studies for the NH_3_-SCR process. In this case ammonia was chemisorbed on the catalyst surface and then its reactivity was analyzed in a flow of gas mixture containing NO and O_2_ with the increasing reaction temperature. The NO conversion started at about 120–140 °C and maximum of NO consumption was observed at about 230 °C for the 25Cu-MCM-41 catalyst (Figure 11A). In general, the maxima of N_2_ and H_2_O evolution are in the similar temperature range as minimum of the NO consumption. The evolution of water vapor at about 100–110 °C is possibly related to evacuation of water molecules physically adsorbed in pores of silicas. The formation of very small amounts of N_2_O, with its evolution maximum at about 210–220 °C, indicates that nitrous oxide is formed as result of the side reaction of NH_3_-SCR rather than direct ammonia oxidation. In the Figure 11A the evolution of ammonia in NH_3_-TPSR (solid line) and NH_3_-TPD (dashed line) was compared. It can be seen that majority of chemisorbed ammonia was consumed in the studied reaction. In the case of iron containing zeolites, 20Fe-MCM-41 (Figure 11B) and 100Fe-MCM-41 (Figure 11C), the NO conversion is observed at higher temperatures. The maxima of N_2_ and H_2_O evolution are located exactly at this same temperature as maximum of NO consumption. It should be noted that the contribution of chemisorbed ammonia molecules converted into reaction products is significantly lower comparing to 25Cu-MCM-41. The formation of N_2_O was not observed in the presence of both Fe-containing catalysts.

**Figure 12:**
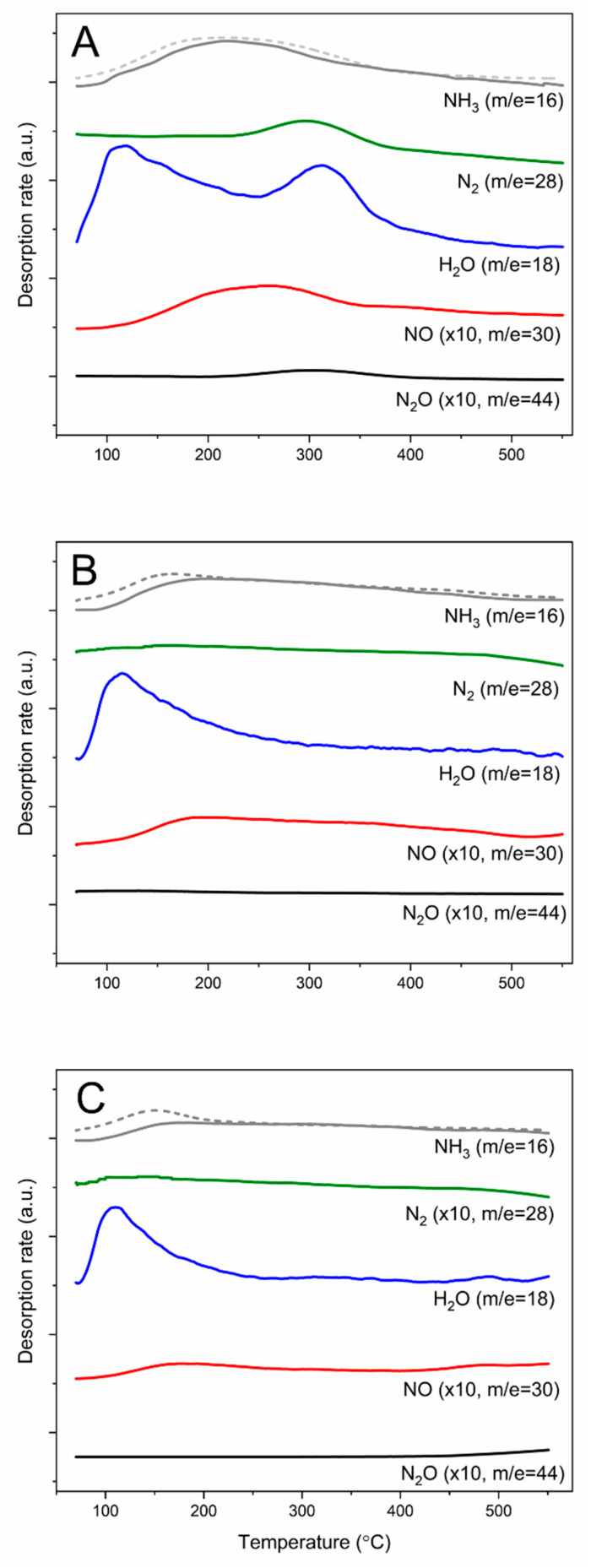
Results of NH_3_-SCO temperature-programmed surface reaction for 25Cu-MCM-41 (**A**), 20Fe-MCM-41 (**B**), and 100Fe-MCM-41 (**C**).

Results of chemisorbed ammonia oxidation (NH_3_-TPSO) are shown in Figure 12. In the case of 25Cu-MCM-41 the evolution of NO is observed at temperature significantly lower than N_2_ evolution (Figure 12A), supporting the concept of i-SCR mechanism, including direct oxidation of ammonia to NO and then the reaction of NO with residual ammonia to N_2_. The small maximum of N_2_O evolution is located in this same temperature range as maximum of N_2_ emission, indicating that nitrous oxide is rather the side product of the NH_3_-SCR process than direct ammonia oxidation. For the iron containing catalysts, 20Fe-MCM-41 (Figure 12B) and 100Fe-MCM-41 (Figure 12C), evolution of small amounts of nitrogen is observed in the broad temperature range, while maxima of the NO production are located at about 190 °C. Thus, in the case of these samples, alternative mechanism of chemisorbed ammonia conversion to N_2_ in the low-temperature range cannot be excluded. The i-SCR mechanism possibly dominates for these catalysts at higher temperatures. It should be noted that only small amount of ammonia chemisorbed on the catalysts is effectively activated to be oxidized by oxygen from gas phase.

## 3. Materials and Methods

### 3.1. Catalysts Preparation

#### 3.1.1. Synthesis of Si-MCM-41 Material

Spherical silica of MCM-41 was synthesized according to the procedure presented by Liu et al. [14]. Cetyltrimethylammonium bromide (CTAB, Sigma-Aldrich, St. Louis, MO, USA), used as surfactant directed agent, was introduced into a solution of aqueous ammonia (Avantor/POCH) and ethanol (Chempur, Karlsruhe, Germany) under stirring. The obtained mixture was stirred for the next 15 min and then tetraethyl orthosilicate (TEOS, Sigma-Aldrich, St. Louis, MO, USA), used as silica source, was added. The synthesis gel, with the molar component ratio of TEOS: 0.3 CTAB: 11 NH_3_: 58 ethanol: 144 H_2_O was aged under stirring continued at room temperature for 2 h. The solid product was separated by filtration, washed with distilled water to obtain pH = 7, air-dried at room temperature for 4 days, and finally calcined at 550 °C for 6 h resulting in spherical MCM-41.

#### 3.1.2. Synthesis of Transition Metal (Cu, Fe) Containing Spherical MCM-41

Cu- and Fe-doped spherical MCM-41 samples were obtained by co-condensation method according to the procedure reported by Szegedi et al. [13].

The spherical Cu-MCM-41 samples, containing copper incorporated to the silica walls, were synthesized by the procedure similar to that used for the synthesis of spherical MCM-41, presented in pervious paragraph. An aqueous solution of CuCl_2_·2H_2_O (POCH, Gliwice, Poland), used as copper source, was added to the aqueous solution containing CTAB, and alcohol, and then the obtained solution was stirred at room temperature for 30 min. The concentrations of copper salt solutions used corresponded to the molar Si/Cu ratios in the final materials of 25 and 50. In the next step, ammonia solution was added, what resulted in the color change of the mixture from light to dark blue. The next steps of the spherical Cu-MCM-41 samples were aged under stirring in the same as in the synthesis of pure silica spherical MCM-41. In the final steps the solid product was separated by filtration, washed with distilled water to obtain pH = 7, air-dried at room temperature for 4 days, and finally calcined at 550 °C for 6 h resulting in spherical 25Cu-MCM-41 and 50Cu-MCM-41, with the intended molar Si/Cu ratios of 25 and 50, respectively.

The synthesis of the spherical Fe-MCM-41 samples with the intended molar Si/Fe ratios of 10, 20, 50, and 100 was done. The required amounts of Fe(NO)_3_·9H_2_O (VWR, Radnor, Pennsylvania, USA), used as iron sources, were introduced into the mixture of CTAB, water, ethanol and TEOS. The obtained light yellow solution was stirred for 30 min, and then, after ammonia introduction, pale beige precipitate was formed. Finally, solid product was separated by filtration, washed with distilled water to obtain pH = 7, air-dried at room temperature for 4 days and calcined at 550 °C for 6 h resulting in spherical 10Fe-MCM-41, 20Fe-MCM-41, 50Fe-MCM-41, and 100Fe-MCM-41, with the intended molar Si/Fe ratios of 10, 20, 50, and 100, respectively.

The detailed information concerning the composition of the reaction mixtures used for the synthesis of spherical MCM-41, *x*Cu-MCM-41, and *x*Fe-MCM-41 are presented in Table 2.

### 3.2. Catalysts Characterization

The X-ray diffraction (XRD) patterns of the studied materials were obtained using a Bruker D2 Phaser diffractometer (Bruker, Billerica, MA, USA). Data were collected in the 2 theta ranges of 1–8° and 28–42° with a step of 0.02°. The counting time of 5 s per step was applied in the range of low-angle XRD measurements, while for the data collecting in the 2 theta range of 28–42° 1 s per step.

The chemical compositions of the samples were determined by the method of inductively coupled plasma optical emission spectrometry (ICP-OES) using an instrument iCAP 7000 (Thermo Scientific, Waltham, MA, USA). The solid samples were dissolved in a mixture containing 6 mL HNO_3_ (67–69%, Honeywell, Charlotte, NC, USA), 2 mL HCl (30%, Honeywell, Charlotte, NC, USA), and 2 mL HF (47–51%, Honeywell, Charlotte, NC, USA) at 190 °C using a microwave digestion system (Ethos Easy, Milestone, Sorisole, Italy). The textural parameters of the samples were determined by N_2_ sorption at −196°C using a 3Flex v.1.00 (Micromeritics, Norcross, GA, USA) automated gas adsorption system. Prior to the analysis, the samples were outgassed under vacuum at 350 °C for 24 h. The specific BET surface area (S_BET_) of the samples was determined using BET (Braunauer–Emmett–Teller) equation. The pore size distributions were calculated from the adsorption branch of nitrogen isotherm by applying BJH (Barrett–Joyner–Halenda) method, while the pore volume was determined by means of the total amount of nitrogen adsorbed at p/p_0_ = 0.98. For the selected catalysts textural parameters were determined also after their squeeze in a hydraulic press with the force of 3 tons.

UV-vis-DR spectra of the samples were recorded using an Evolution 600 (Thermo Scientific, Waltham, MA, USA) spectrophotometer in the range of 200–900 nm with a resolution of 2 nm. SEM images were recorded using Hitachi S-4700 scanning electron microscope (Hitachi Instruments Inc., San Jose, CA, USA) equipped with a Noran Vantage analyzer.

Temperature-programmed desorption of ammonia (NH_3_-TPD) was used for the analysis of the surface acidity of the samples. The measurements were performed in a flow quartz microreactor system equipped with quadrupole mass spectrometer PREVAC-200 (QMS, PREVAC, Rogów, Poland). Prior to the NH_3_-TPD run the sample (50 mg) was outgassed in a flow of pure helium at 550 °C for 30 min. Subsequently, microreactor was cooled to 70 °C and the sample was saturated with ammonia in a flow of gas mixture containing 1 vol.% NH_3_ diluted in helium for about 2 h. Then, the catalyst was purged in a flow of pure helium until the constant base line level was attained. Ammonia desorption was carried out with a linear heating rate of 10 °C/min in a flow of pure helium (20 mL/min). The flow rate of gas mixture was adjusted and controlled by mass flow controllers (Brooks Instruments, 19440-0903 Hatfield, PA, USA).

Calibration of the QMS detector with commercial mixtures allowed recalculating detector signal into the ammonia desorption rate.

The reducibility of the samples was studied by temperature-programmed reduction with using H_2_ as reducing agent (H_2_-TPR). The measurements were carried out in a fixed-bed flow microreactor system equipped with thermal conductivity detector (TCD, Valco, Houston, TX, USA). The flow rate of gas mixture was adjusted and controlled by mass flow controllers (Brooks Instruments, Hatfield, PA, USA). Prior to the H_2_-TPR experiments, each sample (25 mg) was outgassed in a flow of pure argon at 550 °C for 30 min. After cooling down to 100 °C the H_2_-TPR runs were carried out in the range from 100 to 900 °C with the linear heating rate of 10 °C/min in a flow of gas mixture containing 5.0 vol.% H_2_ diluted in argon (flow rate—10 mL/ min).

### 3.3. Catalytic Tests

Catalytic tests in the processes of the selective catalytic reduction of NO with ammonia (NH_3_-SCR) and selective catalytic oxidation of ammonia (NH_3_-SCO) were performed in a fixed-bed quartz microreactor system under atmospheric pressure. The reactant concentrations were continuously monitored using a quadrupole mass spectrometer (PREVAC) connected directly to the reactor outlet. Prior to the catalytic tests, the catalyst samples (100 mg, particle size in the range of 250–315 µm) were outgassed in a flow of pure helium at 550 °C for 30 min. In the case of the NH_3_-SCR process, the gas mixture containing 0.25 vol.% NO, 0.25 vol.% NH_3_ and 2.5 vol.% O_2_ diluted in pure helium (total flow rate of 40 mL/min) was used. While, in the catalytic tests of the NH_3_-SCO process, the reaction mixture containing 0.5 vol.% NH_3_ and 2.5 vol.% O_2_ diluted in helium (total flow rate of 40 mL/min) was applied.

### 3.4. Temperature-Programmed Surface Reactions

Temperature-programmed surface reduction of adsorbed NO with ammonia (NH_3_-TPSR) and temperature-programmed surface oxidation of adsorbed ammonia (NH_3_-TPSO) were performed in the same system as in the case of the NH_3_-TPD experiments. The catalyst sample of 50 mg was outgassed at 550 °C for 30 min, cooled to 70 °C, exposed to the flow of gas mixture containing 1 vol.% NH_3_ diluted in helium and then purged in a helium flow according to the procedure applied in the NH_3_-TPD experiments. In the next step, in the case of NH_3_-TPSR micro-reactor was heated from 70 to 550 °C with the linear heating rate of 10 °C/min in a flow of gas mixture containing 0.25 vol.% NO and 2.5 vol.% O_2_ diluted in helium (total flow rate—20 mL/min). The NH_3_-TPSO runs were conducted in the same temperature range and heating rate but in a flow of gas mixture containing 2.5 vol.% O_2_ diluted in helium (total flow rate—2 mL/min). The reactant concentrations were continuously monitored using a quadrupole mass spectrometer (PREVAC) connected directly to the micro-reactor outlet. For the identification of gas components, the following m/e signals were analyzed: m/e = 16 for NH_3_, m/e = 18 for H_2_O, m/e = 28 for N_2_, m/e = 30 for NO and m/e = 44 for N_2_O. The experiments were carried out for the selected catalysts—25Cu-MCM-41, 20Fe-MCM-41 and 100Fe-MCM-41.

## 4. Conclusions

Spherical MCM-41 containing various content of copper or iron incorporated into silica walls presented a very promising catalytic performance in the NH_3_-SCR process. The increase in transition metal content in MCM-41 resulted in a decreased size of silica spheres and their sticking and therefore limited accessibility of the surface transition metal species. The optimal catalytic performance in a series of iron containing samples presented the catalyst with the intended Si/Fe molar ratio of 20. In this case the NO conversion above 90% with nearly 100% selectivity to nitrogen was obtained in the range of 300–450 °C. The samples with the higher Si/Fe ratio were less active due to lower iron content, while for the catalyst with the lower Si/Fe ratio presented the lowest activity due to sticking of silica spheres and therefore limited accessibility of surface iron centers. On the other side, it seems that catalytic activity of copper containing catalysts in NH_3_-SCR is less sensitive on copper content. The efficiency of the NH_3_-SCR process decreased above 300 °C for the copper containing catalyst and above 375–450 °C for the iron containing catalysts due to the side reaction of direct ammonia oxidation by oxygen present in the reaction mixture. However, also in the high temperature range a very high selectivity to nitrogen was observed. The studies of ammonia oxidation (NH_3_-SCO) showed activity of the copper containing catalyst at temperatures significantly lower than the iron containing samples. Moreover, iron modified silicas presented higher selectivity to N_2_ comparing to copper containing catalysts in the high temperature range. The i-SCR mechanism, as dominating reaction pathways, was postulated in the process of ammonia oxidation.

## Figures and Tables

**Table 1:** Textural parameters, chemical composition and surface acidity of the spherical MCM-41 samples.

Sample Code	S_BET_ [m^2^/g]	PV* [cm^3^/g]	Cu [wt.%]	Fe [wt.%]	Si/Cu-Fe mol/mol	C_a_** [μmol/g]	D_a_*** [μmol/m^2^]	NH_3_/Cu-Fe [mol/mol]
MCM-41	1102	0.769	-	-	-	-	-	-
50Cu-MCM-41	684 (536)	0.443 (0.323)	2.1	-	49.1	152	0.222	0.44
25Cu-MCM-41	441	0.295	5.6	-	17.6	254	0.576	0.35
100Fe-MCM-41	926	0.687	-	1.1	86.2	95	0.103	0.53
50Fe-MCM-41	815	0.648	-	2.2	41.4	145	0.178	0.43
20Fe-MCM-41	939 (991)	0.767 (0.784)	-	4.0	21.4	208	0.222	0.33
10Fe-MCM-41	582	0.789	-	9.8	8.2	292	0.502	0.19

PV*—pore volume; C_a_**—surface concentration of acid sites related to 1 g of the sample; D_a_***—surface density of acid sites—concentration of acid sites on 1 m^2^ of the sample; values in brackets represent S_BET_ and PV of the samples squeezed in a hydraulic press with the force of 3 tons.

**Table 2:** The molar ratio composition of the synthesis mixtures used for the preparation of the transition metal containing MCM-41 microspheres.

Sample Code	TEOS *	CTAB **	NH_3_	C_2_H_5_OH	H_2_O	Cu	Fe
MCM-41	1	0.3	11	58	144	-	-
50Cu-MCM-41	1	0.3	11	58	144	0.02	-
25Cu-MCM-41	1	0.3	11	58	144	0.04	-
100Fe-MCM-41	1	0.3	11	58	144	-	0.01
50Fe-MCM-41	1	0.3	11	58	144	-	0.02
20Fe-MCM-41	1	0.3	11	58	144	-	0.05
10Fe-MCM-41	1	0.3	11	58	144	-	0.1

TEOS*— tetraethyl orthosilicate; CTBA**— cetyltrimethylammonium bromide.
